# Production and Physicochemical Characterization of Gelatin and Collagen Hydrolysates from Turbot Skin Waste Generated by Aquaculture Activities

**DOI:** 10.3390/md19090491

**Published:** 2021-08-28

**Authors:** Jesus Valcarcel, Javier Fraguas, Carolina Hermida-Merino, Daniel Hermida-Merino, Manuel M. Piñeiro, José Antonio Vázquez

**Affiliations:** 1 Group of Recycling and Valorization of Waste Materials (REVAL), Marine Research Institute (IIM-CSIC), Eduardo Cabello 6, 36208 Vigo, Spain; xavi@iim.csic.es (J.F.); jvazquez@iim.csic.es (J.A.V.); 2 Centro de Investigaciones Biomédicas (CINBIO), Departamento de Física Aplicada, Facultad de Ciencias, Universidade de Vigo, 36310 Vigo, Spain; cahermida@uvigo.es (C.H.-M.); mmpineiro@uvigo.es (M.M.P.); 3 Netherlands Organization for Scientific Research (NWO), DUBBLE@ESRF, BP220, F38043 Grenoble, France; daniel.hermida_merino@esrf.fr

**Keywords:** turbot gelatin, aquaculture by-products valorization, marine biomaterials

## Abstract

Rising trends in fish filleting are increasing the amount of processing by-products, such as skins of turbot, a flatfish of high commercial value. In line with circular economy principles, we propose the valorization of turbot skins through a two-step process: initial gelatin extraction described for the first time in turbot, followed by hydrolysis of the remaining solids to produce collagen hydrolysates. We assayed several methods for gelatin extraction, finding differences in gelatin properties depending on chemical treatment and temperature. Of all methods, the application of NaOH, sulfuric, and citric acids at 22 °C results in the highest gel strength (177 g), storage and loss moduli, and gel stability. We found no relation between mechanical properties and content of pyrrolidine amino acids, but the best performing gelatin displays higher structural integrity, with less than 30% of the material below 100 kDa. Collagen hydrolysis was more efficient with papain than alcalase, leading to a greater reduction in Mw of the hydrolysates, which contain a higher proportion of essential amino acids than gelatin and show high in vitro anti-hypertensive activity. These results highlight the suitability of turbot skin by-products as a source of gelatin and the potential of collagen hydrolysates as a functional food and feed ingredient.

## 1. Introduction

Turbot (*Scophthalmus maximus*) is a flatfish of high commercial value, currently produced for the most part in aquaculture farms, mainly in China and Europe [1]. Turbot has been traditionally marketed as a whole fish, but processing companies are increasingly filleting this species driven by consumer demand for convenience food. As this practice expands, so will the amount of waste generated, which needs to be handled to avoid environmental problems and enhance resource efficiency, in line with the principles of the blue economy. 

Probably because of the limited access to turbot by-products so far, very few works have focused on the valorization of this species. A recent study applied proteolysis to turbot frames and trimming, heads, and viscera, recovering a mixture of peptides and amino acids with antioxidant and antihypertensive activities, along with fish oil [2]. In a similar line, another work also obtained antioxidant peptides, this time from the discarded skin of turbot, through fermentative processes [3]. However, collagen is one of the main components of fish skin, and instead of extensive protein breakdown, valuable gelatin can be obtained from this by-product through mild hydrolysis [4].

Gelatin results from the partial hydrolysis of collagen, a structural protein composed of chains of amino acids, mainly glycine, proline, and hydroxyproline, arranged in a triple helix of around 300 kDa. The way these macromolecules self-assemble into aggregates determines the mechanical properties and biological functions of the resulting fibril network [5]. The extremely low solubility of collagen limits its applications, but treatments capable of disrupting the collagen structure render gelatin a more amenable material composed of a mixture of peptides of different molecular weights. In this form, this hydrocolloid has been widely applied in food products, photographic films, cosmetics, drugs, and more recently in the biomedical and tissue engineering fields [4,6,7]. 

Gelatin’s properties must match the intended application and depend mainly on the raw material used and the processing method [8]. Traditionally, cattle bones, hides, and pig skin have been the main sources of gelatin, but religious and socio-cultural reasons have spurred the development of alternative sources, such as insects and fish [9]. Although the rheological properties of fish gelatin cannot match its terrestrial counterparts, gelatin from the skin of warm-water fish and flatfish such as sole and megrim have shown better physical properties than other cold-water fish species [4]. Heat, alkaline and acidic media have proven capable of disrupting the collagen structure in the source material. Temperature, pH, reaction time, and ionic strength influence the process and hence the quality and properties of the resulting gelatin [10,11].

In the present work, we characterize for the first time gelatin from turbot skin under the hypothesis that its rheological properties may be in line with other flatfish, applying several extraction methods to determine the most suitable for turbot. The characterization includes the determination of absolute molecular weights of gelatin distributions by gel permeation chromatography with light scattering detection, secondary structure by infrared spectroscopy, and associated rheological properties. To complete the valorization process, the material remaining from gelatin extraction is enzymatically hydrolyzed and characterized.

## 2. Results and Discussion

### 2.1. Gelatin Characterization

#### 2.1.1. Yield

In the present work, we report for the first time the valorization of skin, generated as waste in the filleting of aquaculture turbot, as a source for the production of gelatin and collagen derivatives, following the principles of sustainability and bioeconomy. As a first step, this study aims to evaluate different chemical methods and temperature conditions to maximize the extraction of high-quality gelatin. The yield of gelatin, calculated as dry weight of gelatin extracted per 100 g of fresh skin before processing, ranges between 4.5 and 9.1% for M6 and M4, respectively (Table 1). As expected, the application of different treatments and chemicals on fish skin leads to significant differences in the amount and characteristics of gelatin extracted, in line with previous reports on other fish [12,13,14]. In all methods, the best temperature to recover the largest amount of gelatin is 4 °C (*p* < 0.05). M2 and M4 show the highest yields without significant differences among them (*p* > 0.05). Similar behavior occurred in the production of gelatin from pollock skin [15], in which less gelatin was lost at a cool temperature in comparison with room temperature processing. No significant differences (*p* < 0.05) exist between the complete alkaline-acids treatments at 22 °C (M1) and the ones only based on a citric acid procedure at the same temperature (M3). Identical behavior appears in the comparison between M2 and M4. Nevertheless, the highest quantity of gelatin extracted from turbot (9% *w/w*) was inferior to that reported for skins of tuna (13% *w/w*) [8]. 

Additionally, the proximal composition of dry gelatins ranged in the following intervals: moisture (5–8%, obtaining the minimum and maximum values in M7 and M5), ash (1.9–8.9%, with M2 and M3 showing the lowest and highest figures), total lipids (1.6–4.5%, with M6 and M4 as the minimum and maximum percentages) and total protein (80–91%, observing the minimum and maximum content in M3 and M7).

#### 2.1.2. Gel Strength

In terms of applicability, the strength of gelatin gels is one of the physical properties most appreciated since it is the parameter that mainly defines the quality of this biopolymer [16]. Table 1 summarizes gel strength measurements, revealing significant differences (*p* < 0.05) between pairs of identical treatments (M1/M2 and M3/M4) at different temperatures. Operations conducted at 22 °C led to higher gel strengths, and conversely, to lower gelatin yield. Gelatin solutions obtained by the phosphoric acid method, independently of temperature processing, did not jellify, and the values of gel strength were therefore null. This result is contradictory with the assumption of Jamilah and Harvinder (2002) [17] that direct extraction of gelatins in a weak acid, such as phosphoric acid, without previous alkaline and acid pre-treatments protects the loss and hydrolysis of collagen material in each sequential wash. In fact, the method to produce gelatin of the best quality in terms of gel strength is M1, which includes an alkaline and two acidic treatments before thermal extraction of the gelatin solution. This procedure was even better (177 g) than using only one acid wash with citric acid (132 g) and also than the protocol incorporating saline treatment (82 g). Other authors have mentioned that sodium chloride destabilizes and breaks different bonds of gelatin structure [18], which could explain the present results.

Our data of turbot gelatin (177 g) is slightly lower than those previously obtained in our lab from blue shark and tuna skin wastes [8] and also lower than commercial pork and bovine gelatins (200–300 g). This bloom value lies halfway between gelatin from warm-water species (grass carp and tilapia: 291–328 g) [19,20] and cold-water fish (Atlantic cod and salmon: 70–108 g) [21,22].

#### 2.1.3. Amino Acid Composition

Table S1 (supplementary material) summarizes the profiles of amino acids present in turbot gelatin. Gelatin, as a hydrolyzed protein derived from collagen, must contain the most predominant aminoacids in such triple helical protein, glycine, proline, and, above all, hydroxyproline that is exclusive to collagen. In all cases, glycine is the most relevant amino acid in terms of percentage, ranging from 20.9% in M5 to 22.1% in M3, but with no significant differences among extraction methods (*p* > 0.05). 

Considering the total protein content in the organic matter of dry gelatin, as a sum of amino acids, such levels are superior to 90%. In all gelatin samples, the sum of Pro + OHPro, is higher than 17.3%, reaching up to 19.3–19.4% in M2 and M1. Based on this data, the differences in processing do not seem to affect OHPro content, as was reported by Nikoo et al. (2013) [23], but the sum of pyrrolidine amino acids is lower in phosphoric acid treatments. Neither OHPro nor total pyrrolidine amino acid content seem to correlate with gel strength (Table 1). This is in contrast with previous reports linking the presence of pyrrolidine amino acids to the formation of hard and stable gels due to the ability of –OH groups in OHPro to form hydrogen bonds, hence helping in the stabilization of the triple-helical skeleton of the gelatin extracted [24,25].

In general, the amino acid profiles are in concordance with gelatin generated from Alaska pollock [26], blue shark [8], and codfish [22], but as expected, imino acid content is lower (19.4%) than in pork skin gelatin (22.3%) [27]. Additionally, the ratio among essential and total amino acids (TE/TA) does not exceed 26%. This percentage, although valuable, is lower than that reported in fish protein hydrolysates or fish proteins that are considered an excellent source of essential amino acids for human nutritional supplements [28,29].

#### 2.1.4. Molecular Weight Distribution Profiles

**Figure 1:**
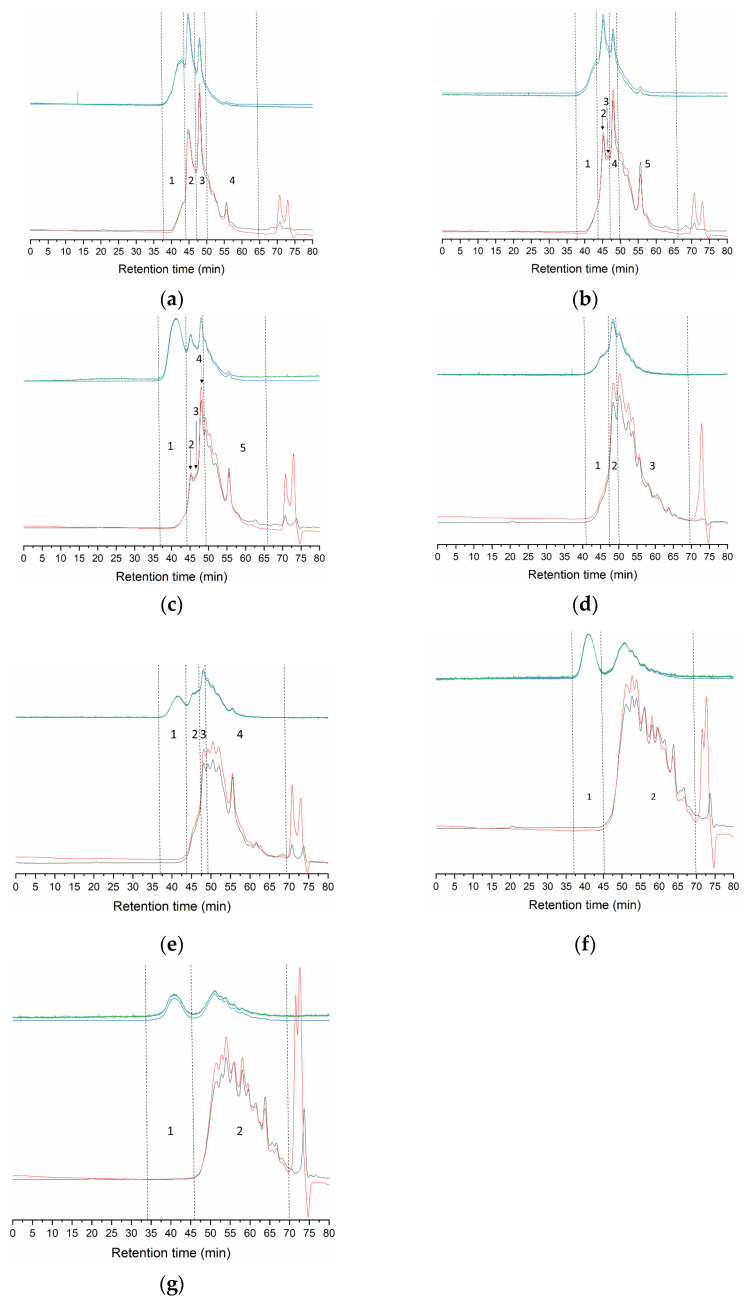
GPC eluograms of turbot gelatin extracted by different methods. (**a**): M1; (**b**): M2; (**c**): M3; (**d**): M4; (**e**): M5; (**f**): M6; (**g**):M7. Blue line: right angle light scattering; green line: low angle light scattering; red line: refractive index; black line: ultraviolet (232 nm). Detailed information of the numbered regions can be found in Table 2.

The GPC distribution profiles shown in Figure 1 consist of a series of overlapping peaks with different patterns depending on the extraction method but sharing some common features. The first eluting peaks start at around 35 min and are characterized by a low concentration (0.6–7.9% of the total refractive index area, Table 2), associated with intense signals in light scattering, indicative of high molecular weight species. The actual molecular weight of this fraction cannot be accurately calculated but might correspond to tropocollagen and higher molecular weight aggregates. Next, distinct peaks arise at 45.3–45.6 min in samples M1-M3, with estimated molecular weights around 200 kDa, which probably correspond to β-chains (cross-links between two α-chains). Additionally, in M2 and M3, a small fraction of around 150 kDa appears at 47.5–47.6 min, maybe breakdown products of β-chains or other aggregates. Curiously, in M4 and M5, the estimated Mw for peaks also appearing at 47.5–47.6 min reach around 200 kDa. While this Mw is typical of β-chains, the fractions elute two minutes later than the better-preserved profiles seen for M1-M3 (Figure 1); therefore, the material constituting these peaks might be more similar to the 150 kDa fraction in M2 and M3. In all samples, except for M6 and M7, Mw estimates of peaks eluting at 48.4–48.9 min are slightly above 100 kDa, which correspond to α-chains. Finally, the last eluting fraction consists of several overlapping peaks of less than 100 kDa, possibly degraded α-chains and other non-collagenous peptides. 

In general, methods capable of preserving collagen structural units lead to gelatin with higher gel strength (Table 1). In M1 (177 g), β- and α-chains comprise 71.4% of the material, with only 29% of low Mw fragments (Table 2). On the other extreme, skins treated only with phosphoric acid (M6 and M7) released a material fundamentally composed of peptides below 100 kDa (99%), with null gel strength. Remarkably, M2 and M3 show a similar percentage of α- and β-chains and high Mw species (Table 2) but significantly different gel strength (91 and 132 g, respectively). The one possibly important difference lies in the first eluting fraction. Even though accurate Mw estimates are not possible, the considerably higher intensity of the light scattering signal in M3 than in M2 (Figure 1) at similar concentrations indicates higher Mw of this fraction in M3. M4 and M5 show looser structuring, with a high percentage of low Mw species (72–83%), accompanied by lower gel strength (60 and 81 g, respectively). Although lower, the differences with M3 (91 g) were not statistically significant, despite the quite different Mw distribution profiles.

Comparison of Mw distributions of M1 vs. M2 and M3 vs. M4 indicates that extraction at 22 °C better preserves collagen structural units than treatments at 4 °C. Accordingly, sequential treatment with sodium hydroxide, sulfuric and citric acids at room temperature (M1) releases gelatin with the lowest percentage of low Mw species, followed by the same procedure at 4 °C (M2), which is similar to extraction at room temperature with only citric acid (M3). Lowering the temperature maintains the same proportion of α-chains as in M2 and M3, accompanied by a lower amount of higher Mw species (M4). Sequential cold treatment with NaCl and NaOH followed by acetic acid at 22 °C (M5) does not improve the Mw profile, and warm phosphoric acid after both cold and room temperature treatment with NaOH results in the almost complete destruction of collagen structural units (M6 and M7), to the point that temperature produces no effect.

#### 2.1.5. Thermal Stability

**Figure 2:**
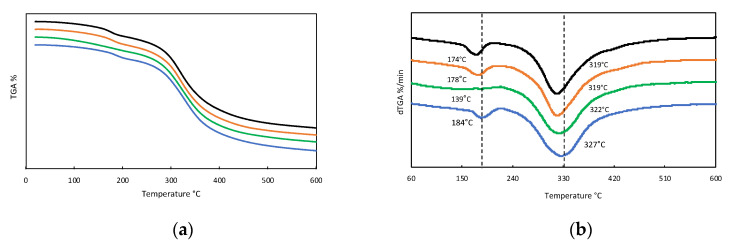
Stacked TGA (**a**) and dTGA (**b**) thermograms of turbot gelatin. M1 (blue line **—**), M2 (green line **—**), M3 (ochre line —), and M5 (black line —). Temperature values correspond to minima in each sample.

We selected four gelatins for further study (M1, M2, M3, and M5) based on the results of gel strength and molecular integrity determined by GPC. As a starting point, we were interested in assessing the thermal stability of the gelatin samples. The TGA thermogram in Figure 2 shows similar profiles of weight loss with increasing temperature for all samples that can be divided into two separate regions. First, a slight degradation step with onset at around 160 °C, except for M2 that declines smoothly and shows no inflection point in the first derivative plot (Figure 2b). Nevertheless, this first stage leads to a weight variation of around −10% for all samples due to loss of absorbed water [30]. Second, a much more pronounced decline in a mass close to 300 °C, carrying a 60% weight reduction. This stage corresponds to the degradation of the low molecular weight protein fraction, as well as structurally bound water [31].

#### 2.1.6. Infrared Spectroscopy

**Figure 3:**
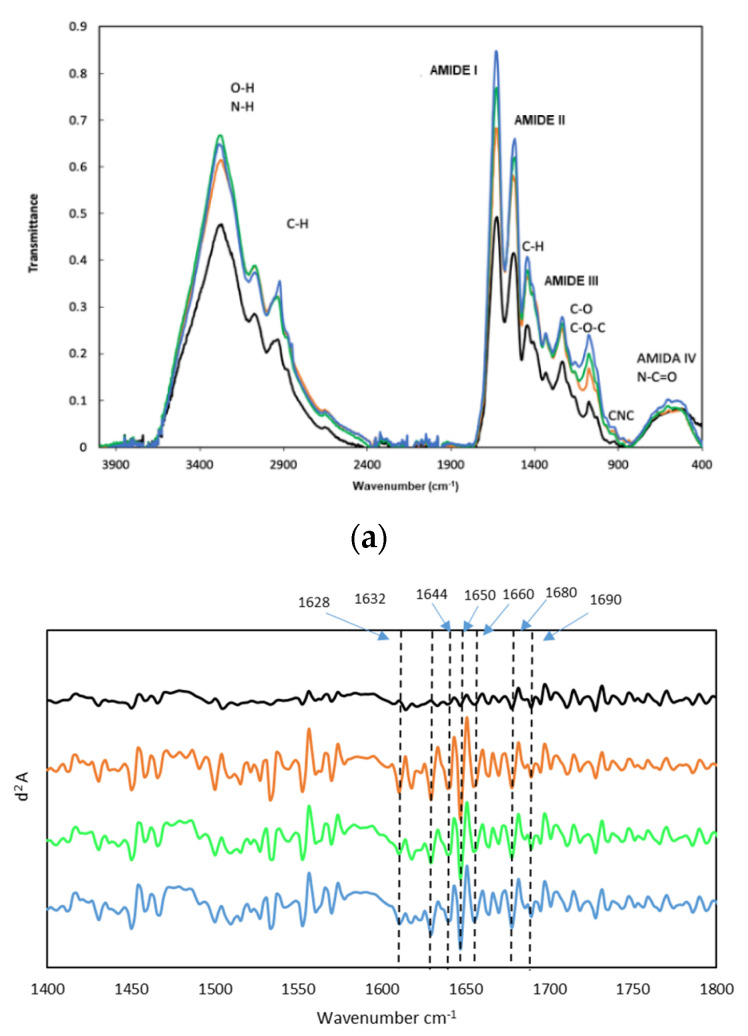
FTIR spectra of turbot gelatin (**a**) and spectra of the second derivative (differential FTIR spectra, with FDD method in the absorption region of amide I (**b**): (—) M1, (—) M2, (—) M3, and (—) M5.

The FTIR spectra displayed in Figure 3a show the characteristic bands of gelatin, with wavenumbers associated with maxima in Table S2. The spectra profiles are typical of fish gelatin and comparable to those reported for other fish species [32,33,34]. At high wavenumbers, an intense and broad band with maxima at 3277–3286 cm^−1^ appears, formed by contributions by the stretching modes of the N-H bonds of protein and O-H groups of carbohydrates and water. The band at 3070–3076 cm^−1^ is also due to N-H bond tension modes. Signals between 2850 and 3000 cm^−1^ correspond to the tension modes of the C-H bonds of aliphatic chains, with bending modes at 1355 and 1445 cm^−1^.

Amide bands are closely related to the secondary structure of gelatin, mainly bands I (1627–1631 cm^−1^), due to the stretching mode of the CO bond of the peptide bonds, II (1520–1531 cm^−1^), due to bending of the N-H bonds and tension of the C-N bonds, and III (1236–1238 cm^−1^), due to C-N stress together with the NH bends, and a small contribution from the C-C stress and the in-plane bending CO. Intensity of these bands have been associated with changes in structural order [35]. In turbot gelatin, the intensity of amide bands I-III decreases in the following order: M1 > M2 > M3 > M5, showing M5 the largest reduction (Figure 3a) which probably implies loss of secondary structure from M5 trough to M1. This agrees with the proportion of high Mw species determined by GPC (M1 7.9%; M2 4.7%; M3 3.8%; M5 0.6%), most likely containing species in triple helix conformation and higher aggregation states.

Other less intense bands emerge at 540–551 cm^−1^ (amide IV) due to the bend of O=C-N groups; at 600–654 cm^−1^ (amide V), due to N-H bends; and at 700–708 cm^−1^ (amide VI). At 923–926 cm^−1^, a weak band appears corresponding to the symmetric tension mode of the CNC link. The bands observed at 1161, 1077, and 1031 cm^−1^ are due to different tension modes of C-O and C-O-C bonds that could be due to carbohydrates. Slight displacements (~2 cm^−1^) are observed between the different methods of extraction of Turbot gelatin (Table S2).

Application of the second derivative to amide band I, the most informative about the secondary structure of the protein, allows for segregating the band into several peaks (Figure 3b). A number of previous works have assigned the wavenumbers of these peaks to secondary structure [36,37,38,39,40], as displayed in Table 3. Second derivative spectra show similar profiles for M1, M2, and M3, but quite different to M5, with associated lower intensity in the latter (Figure 3b). This difference probably reflects the lower gel strength and molecular weight reported above. The most intense signal at 1650 cm^−1^ corresponds to random coil disposition, indicating the predominance of disordered structure in samples M1 to M3. Components at 1628 and 1632 cm^−1^ have been attributed to hydrated imides with some contribution from the β-sheets; the component at 1680 cm^−1^ is defined by the presence of β-turns, and the component at 1690 cm^−1^ has been assigned to the β-sheets with some contribution from the β-turn absorbance (Table 3). Nevertheless, these gelatins also contain helical structures associated with signals of significant intensity at 1660 cm^−1^. 

#### 2.1.7. Viscoelastic Properties

**Figure 4:**
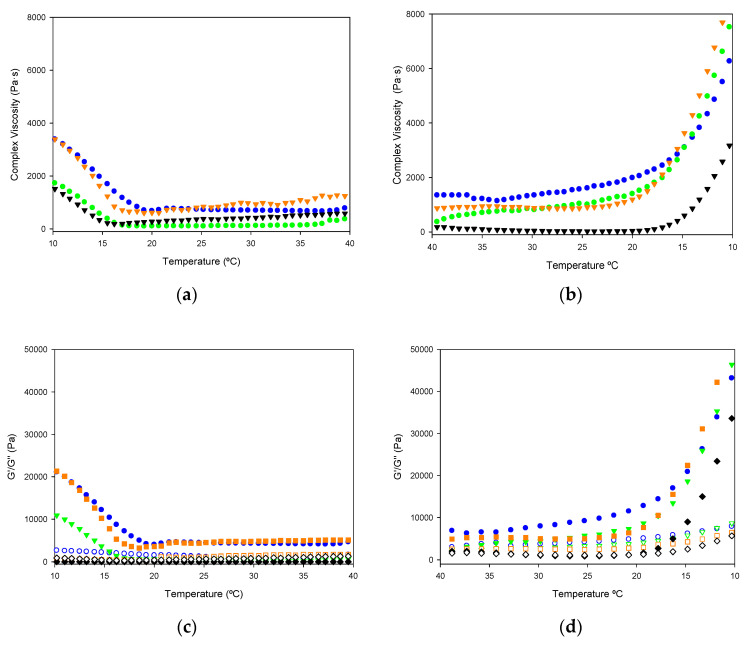
Variation of complex viscosity (**a,b**); storage modulus (G′, filled circle) and loss modulus (G″, empty circle) (**c,d**); for 30% solutions of gelatin hydrogels with temperature. Heating ramp (left column) from 10 to 40°C, cooling ramp (right column) from 40 to 10 °C. M1 ((●) blue color), M2 ((●) green color), Extraction M3 ((●) orange color), and M5 ((●) black color).

As a starting point in assessing viscoelastic properties, rheological measurements of gelatin solutions upon heating and cooling provide information about gelling kinetics, namely complex viscosity, storage and loss moduli, damping factor, and phase angle (Figure 4).

As can be seen in Figure 4a, the complex viscosity of samples M1 and M3 is higher compared to M2 and M5, in striking difference with superior gel strength in M1 and M3 as seen above. For all samples, a transition in complex viscosity occurs around 15 °C (Figure 4a,b). In the heating ramp, after the peak at 11 °C, a rapid decrease in complex viscosity up to 20 °C is followed by a linear behavior, suggesting that the network structure formed during gelatinization was disrupted upon temperature increase. Similar viscosity versus temperature behavior has been observed for gelatin fish [41].

However, the values of the storage modulus (G’) were found higher than the loss modulus G’’ (Figure 4c,d) for gelatins obtained by all extraction methods, indicating that the elastic behavior of the system is greater than the viscous behavior, and hence the formation of a large elastic network. The pattern of variation of the storage modulus with temperature is virtually identical to that of complex viscosity, showing M1 the highest values, followed by M3, with M2 and M5 at a considerable distance. In the heating ramp, M1, M3, and to a lesser extent, M2 show a decrease in both moduli (Figure 4c) between 10 and 20 °C, representing the transition from gel to solution state. In the cooling ramp, M1 produces a more pronounced peak for G’/G’’ (Figure 4d) compared to the other extraction methods with an increase in both modules around 18 °C. The increase in G′ upon the cooling process is related to the transition from solution to gel state caused by triple-helix formation.

In the temperature range of 15–5 °C in the cooling ramp, higher values of the storage and loss modules are obtained, as well as the complex viscosity, so the process is not reversible; perhaps due to evaporation of the sample since it is the lowest temperature at which the equipment can measure.

The strain sweeps allow evaluating the viscoelastic behavior of gelatins by determining the range where their rheological properties are independent of the applied deformation. The four samples with the highest gel strength value were analyzed to compare the deformation-structure relationship at 20 °C.

**Figure 5:**
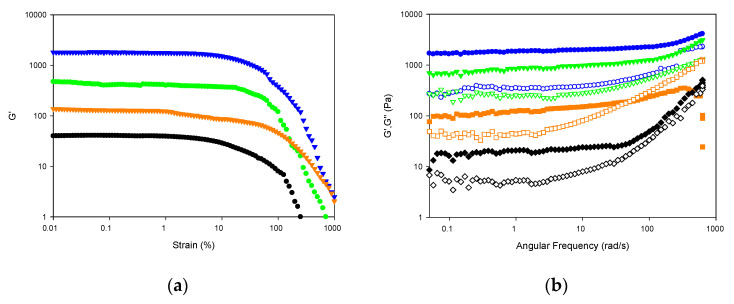
Strain and frequency sweep for 30% solutions of turbot gelatin. Store (G’; filled symbols) and loss moduli (G’’; hollow symbols) depicted versus strain (**a**) and angular frequency (**b**). M1 (blue); M2 (green); M3 (ochre); M5 (black).

Strain scanning of the 30% gelatin gels has shown that the viscoelasticity remains linear up to 0.1% strain for all extraction methods used (Figure 5a). The system becomes solid, characterized by increasing G′ and fluidizing at lower stresses [42].

An oscillatory frequency sweep to understand the dynamic behavior of the gelatin network. A parallel storage and loss modulus profile of the frequency sweep from 0.1 s^−1^ to 10 s^−1^, flowing homogeneously (0.1% 0.05 and 600 rad/s at 20 °C), confirming the network of gel (Figure 5b).

M1 shows the highest value for the storage and loss modulus; for M5 and M3 at higher frequencies, there is a crossing of G’’ over G’, this means that another relaxation zone begins, which approaches the transition of the colloidal gel and therefore this large increase in G’’, indicating higher gel strength [43].

#### 2.1.8. SEM

**Figure 6:**
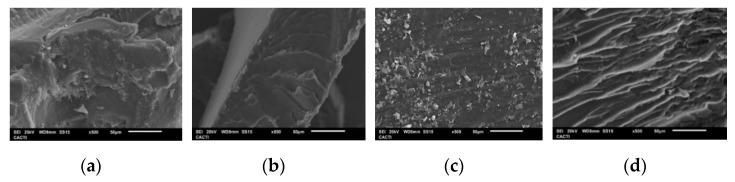
SEM images of different extraction methods of Turbot gelatin: M1 (**a**), M2 (**b**), M3 (**c**), and M5 (**d**).

Figure 6 shows the microscopic structures of gelatin visualized by SEM (Figure 6). All samples display a lamellar structure, but M5 exhibits a looser network with fairly uniform strands compared to the other extraction methods. This difference may be related to more limited structuring at the molecular level, as shown by the higher proportion of peptides below the Mw of α-chains (100 kDa) determined by GPC, compared to the other samples.

### 2.2. Production of Collagen Hydrolysates from Skin Waste after Gelatin Extraction

In order to reach the complete valorization of turbot skins, the rest of the skins produced as final wastes after aqueous gelatin extraction (SR) were subsequently used as a substrate for the production of enzymatic hydrolysates rich in collagen residues. The proximal composition of the SR was as follows: 80.8 ± 12.6%, 17.9±12.2%, 1.25 ± 0.47%, 24.4 ± 1.5%, and 74.6 ± 4.2%, for moisture, organic matter, ash, total lipids, and total protein. We tested and compared two well-known proteases (alcalase and papain), working at two reaction times, for the generation of the hydrolysates derived from collagen material (CH1-CH4). Both enzymes operated at the corresponding optimal values reported in previous works for the digestion of different fish waste [2,44].

**Figure 7:**
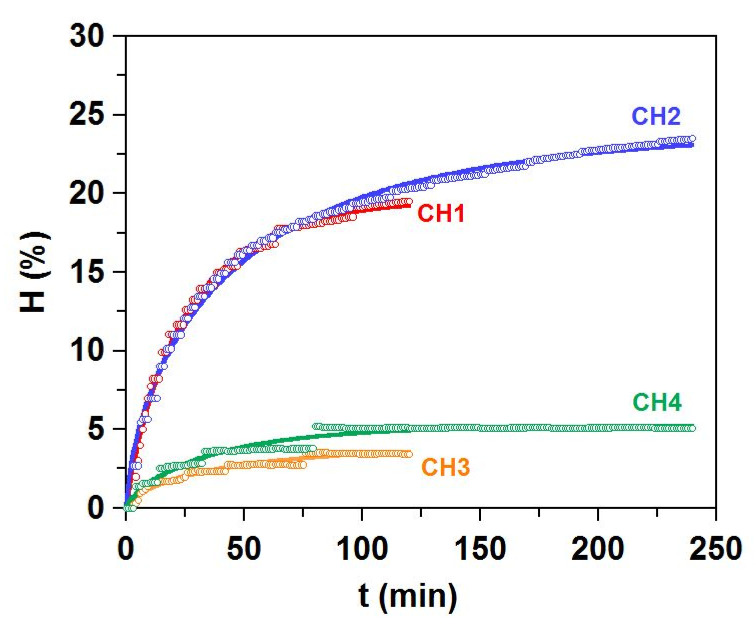
Proteolysis kinetics of SR by alcalase (CH1 and CH2) and papain (CH3 and CH4). Experimental data of hydrolysis degree (H, symbols) were fitted to the Weibull equation (continuous line).

Figure 7 shows the time-evolution of SR hydrolysis and the corresponding simulations obtained by the Weibull equation. The correlation among both data, predicted and experimental, is perfectly described by the mentioned non-linear equation (R^2^ 0.944–0.993). Additionally, the robustness of fittings is also demonstrated by the Fisher F-test (*p* < 0.005, values not shown). As expected, the maximum degrees of hydrolysis (*H_m_*) are significantly higher at longer proteolysis kinetics (*p* < 0.05), and alcalase leads to larger values of *Hm* and *vm* in comparison to papain (Table 4).

In agreement with these outcomes, the highest efficiency of alcalase is confirmed by the yields of digestion, the soluble protein released, and the percentage of non-digestible skin: greater and lower values of Y_dig_, Pr, and Y_skin_, respectively, are provided by alcalase. Based on a similar pH-stat reactor processing, alcalase proteolysis of heads, trimmings, frames, and viscera from turbot achieved levels of *Hm* around 28–35% [2]. 

The most predominant amino acids present in the collagen hydrolysates are also Gly and Glu (Table 4); however, the % of Gly was significantly inferior to the corresponding gelatin. The presence of Pro and OHPro is also lower than observed in Table S1, but the ratio of essential amino acids is healthier in hydrolysates (35–39%) than in gelatins (25–26%) (Table 4). Finally, the bioactivities of hydrolysates are dependent on the enzyme and time applied. CH2 (alcalase-4 h) shows the best results in both in vitro assays conducted, digestibility and antihypertensive action, highest Dig and I_ACE_ values, and lowest IC_50_ data. This result is especially valuable since it represents more than a 5-fold and 10-fold increase over the antihypertensive capacity of previous hydrolysates produced from turbot [2] and salmon by-products [29], respectively. 

**Figure 8:**
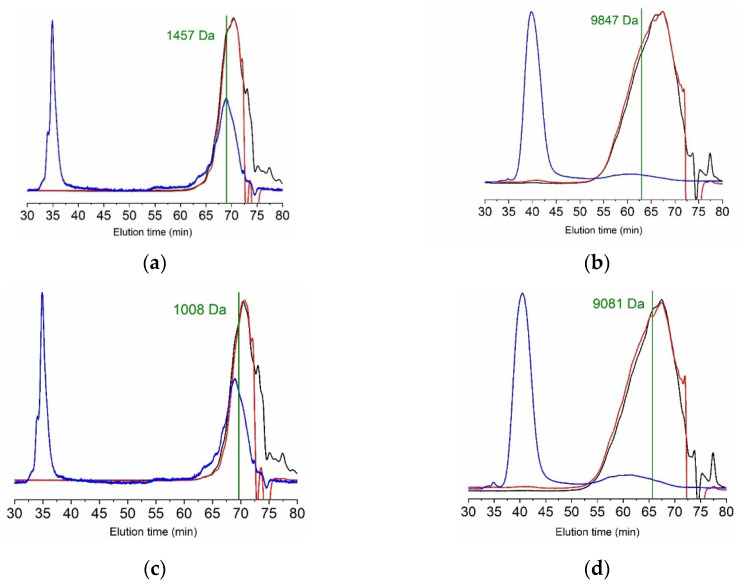
GPC eluograms of hydrolysates from turbot skin after gelatin extraction by different methods. (**a**): Alcalase 2 h (CH1); (**b**): Alcalase 4 h (CH2); (**c**): Papain 2 h (CH3); (**d**): Papain 4 h (CH4). Blue line: right angle light scattering; red line: refractive index; black line: ultraviolet (232 nm).

Molecular weight estimations of collagen hydrolysates reflect the differences seen in proteolysis kinetics. The higher efficiency of alcalase compared to papain results in hydrolysates with Mw of 1.0–1.3 kDa in the first case versus 8.7–10 kDa in the latter (Table S3). Longer reaction times lead to lower Mw with both enzymes, as expected. Furthermore, the polydispersity of papain hydrolysates was greater than for alcalase, producing broader peaks, as seen in Figure 8. Besides the main peak with a maximum at around 70 min, another very intense signal in the light scattering detector appears at 35 and 40 min for alcalase and papain, respectively. Early elution and intensity indicate high molecular weight species; however, its concentration is very low, especially in alcalase samples, as shown by the refractive index and UV detectors.

Digestibility and antihypertensive activity (Table 4) appear correlated with the Mw of the hydrolysates, with more fragmented protein possessing higher digestibility and antihypertensive activity. However, previous studies have reported contradictory results, finding direct [45], inverse [46], or no relationship [29,47].

## 3. Materials and Methods

### 3.1. Skin By-Products from Aquaculture Fish

Fresh skins generated as by-products in the filleting of turbot (*Scophthalmus maximus*) from aquaculture were kindly provided by Prodemar (Stolt Sea Farm S.A., Carnota, A Coruña, Spain). The turbots used for filleting were always adult individuals with sizes of around 2–3 kg and with ages of 24–30 months. Skins were stored at −20 °C until they were processed by cutting into 5 cm squares maximum (500 g processed per batch). In all cases, the skin fragments were washed with water for 30 min under orbital agitation (50 rpm) to eliminate impurities.

### 3.2. Production of Turbot Gelatin

Different methods of gelatin extraction were evaluated (Figure S1). The first methods (M1 and M2) included in the first stage three sequential chemical treatments [8] at 22 °C and 4 °C, respectively, using: (a) 0.05 M NaOH with a solid:liquid ratio of (1:4) for 30 min under 50 rpm of orbital agitation; (b) 0.02 M H_2_SO_4_, (1:4 ratio), 30 min and 50 rpm; (c) 0.052 M citric acid, (1:4 ratio), 30 min and 50 rpm. Between each treatment, a water washing step was applied for 30 min. In methods M3 and M4, only the citric acid treatment (in the same conditions indicated previously) was run at 22 °C and 4 °C, respectively.

The sequential stages applied in method 5 (M5) followed recommendations previously reported [24,48]: (a) an initial 0.8 M NaCl treatment at 1:4 ratio for 30 min at 4 °C and under 50 rpm of agitation; (b) a procedure using 0.2 M NaOH, 1:6 ratio, 30 min, 4 °C, 50 rpm, and repeating this step three times; (c) a procedure using 0.05 M acetic acid, 1:10 ratio, 3 h, 22 °C, 50 rpm. Again, water washing (1:4 ratio) for 30 min was performed in between steps.

Methods 6 and 7 (M6 and M7) were based on the thermal extraction of gelatin in acidic conditions [49]: (a) first, treatment with 0.1 M NaOH, (1:2 ratio), 30 min, 50 rpm at 22 °C for M6 and 4 °C for M7; (b) H_3_PO_4_ solution until pH 5–5.2, (1:2 ratio), 3 h, 50 rpm at 50 °C for both protocols.

The elimination of the chemical effluents in each procedure (alkaline and acidic effluents) was carried out by filtration (1000 µm). At the end of the chemical treatments, gelatin was thermally extracted in all methods at 45 °C on an aqueous medium (1:2 ratio) for 16 h, except for M6 and M7. Afterward, gelatin solutions were purified by filtration (500 µm), active charcoal adsorption (at 1.5% *w*/*v* for 2 h), and centrifugation (15,000× *g*/20 min). The clean supernatants derived from this last procedure were finally oven-dried for 48–72 h to obtain solid gelatin. In all cases, each method (M1–M7) was executed in duplicate.

### 3.3. Production of Collagen Hydrolysates (FPH)

The remaining solids after the thermal extraction of gelatin from all methods were pooled, split into 1 kg aliquots, ground, and then hydrolyzed by two commercial proteases: Alcalase 2.4L (Novozymes, Nordisk, Bagsvaerd, Denmark) and Papain 6000 (Gygyc Biocon, Barcelona, Spain). The experimental conditions of hydrolysis were (a) 0.2% alcalase (*v*/*w*), 60.3 °C, pH 8.82, t = 2 h of hydrolysis (CH1); (b) 0.2% alcalase (*v*/*w*), 60.3 °C, pH 8.82, t = 4 h of hydrolysis (CH2); (c) 0.2% papain (*w*/*w*), 65 °C, pH 7, t = 2 h of hydrolysis (CH3) and (d) 0.2% papain (*w*/*w*), 65 °C, pH 7, t = 4 h of hydrolysis (CH4). All reactions were performed in a 5 L glass-reactor (pH-Stat system equipped with additional temperature, agitation, and reagent-addition control), mixing 1 kg of skin remains in 1 L of distilled water. A solution of 5 M NaOH was used for pH control, while agitation was continuously maintained at 200 rpm. At the end of the enzymatic digestion process, non-hydrolyzed materials (scarces) were removed by filtering, and the liquid fraction was centrifuged (15,000× *g* for 20 min) to separate oil and hydrolysates. These hydrolysates were immediately warmed (90 °C/15 min) for proteases inactivation. The hydrolysis degree (*H*, as %) was calculated according to the pH-Stat method, and mathematical equations previously described [50,51]. The Weibull equation was applied to predict the experimental kinetics of H [52]:

(1) $$H=H_{m}\{1-\exp[-\ln2{\left(\frac{t}{\tau }\right)}^{\beta }]\}$$

(2) $$v_{m}=\frac{\beta H_{m}\ln2}{2\tau }$$

where *H* is the hydrolysis degree (%), t is the hydrolysis time (min), *H_m_* is the maximum hydrolysis degree (%), β is a dimensionless parameter associated with the slope of the hydrolysis process, *v_m_* is the maximum hydrolysis rate (% min^−1^), and *t* is the time needed to reach the semi-maximum hydrolysis degree (min). The yield of digestion (Y_dig_) of the raw material to the liquid phase was also calculated (in %) [52].

### 3.4. Characterization

#### 3.4.1. Chemical Composition and Bioactive Properties

The chemical composition of turbot gelatin and skin remains were obtained by quantifying: (1) moisture, organic matter, and ash percentage [53], (2) total protein as total nitrogen × 6.11 [8], and (3) total lipids [54]. The amino acid content in gelatin was also quantified by the ninhydrin reaction [55] using an amino acid analyzer (Biochrom 30 series, Biochrom Ltd., Cambridge, UK) and norleucine as an internal standard. In collagen hydrolysates, the following was determined: (1) total soluble protein by the Lowry method [56], (2) in vitro digestibility by the AOAC Official Method according to the reformulations suggested by Miller et al. [57], and (3) in vitro antihypertensive activity Angiotensin I-converting enzyme (ACE) inhibitory activity (I_ACE_) calculating IC_50_ values (protein-hydrolysate concentration that generates a 50% of I_ACE_) by dose-response modeling [58,59]. All analyses were carried out at least in duplicate.

#### 3.4.2. Gel Strength

The strength of the turbot gelatin was measured as previously specified [60]. Briefly, solutions of gelatin were prepared at a concentration of 6.67% (*w/v*), completely dissolved at 45 °C, and cooled at 4 °C for 16–18 h [48]. Gel strength was measured using a Stevens-LFRA Texture Analyzer (Hucoa Erlöss S.A., Madrid, Spain) with a 1000 g load cell equipped with a 0.5 inch diameter Teflon probe. A trigger force of 5 g and a penetration speed of 1 mm/s were used, and gel strength was expressed as maximum force (in g), taken when the plunger had penetrated 3 mm into the gelatin gels, as an average of three determinations.

#### 3.4.3. Molecular Weight

The molecular weight of both gelatin and collagen hydrolysates was estimated by gel permeation chromatography with an Agilent 1260 system equipped with quaternary pump (G1311B), injector (G1329B), column oven (G1316A), diode array (G1315C), refractive index (G1362A) and dual-angle static light scattering (G7800A) detectors. Separation was achieved with a set of four columns (Proteema, PSS, Mainz, Germany): precolumn (5 µm, 8 × 50 mm), 100 Å (5 µm, 8 × 300 mm), 300 Å (5 µm, 8 × 300 mm), and 1000 Å (5 µm, 8 × 300 mm). Samples were eluted at 20 °C with 0.15 M sodium acetate: 0.2 M acetic acid, pH 4.5 at 0.5 mL/min. Samples were dissolved in the mobile phase at 2 g/L. The detectors were calibrated with a polyethylene oxide standard (PSS, Mainz, Germany) of 106 kDa (Mw) and refractive index increment (dn/dc) of 0.135. For gelatin samples, molecular weights were calculated with a dn/dc of 0.190 [61], applying a dn/dc for protein hydrolysates of 0.185 [47].

#### 3.4.4. Infrared Spectroscopy

Fourier transformed infrared spectroscopy by attenuated total reflectance (ATR-FTIR) was obtained using a Spectrometer Nicolet 6700 (Thermo Fisher Scientific, Waltham, MA, USA), equipped with a source IR -Turbo fitted with a detector DTGS in a beamsplitter of KBr. Background scans were 34 with a spectral resolution of 4 cm^−1^ at ambient temperature, using an Attenuated Total Reflectance (ATR) accessory. Gelatin samples were deposited in a gold support in a humid chamber to avoid evaporation during measurements. A study of the second derivative of the spectra of Amide I was carried out using the first difference derivative (FDD method).

#### 3.4.5. Thermogravimetric Analysis

TGA measurements were performed with a Setsys Evolution 1750 Simultaneous Thermogravimetric Analysis (TGA)/Differential Scanning Calorimetry (DSC) instrument (Setaram, Caluire-et-Cuire, France), presented in previous publications [61]. About 12 mg of samples were introduced in a sealed capsule, undergoing temperature sweeps from room temperature to 600 °C at a heating rate of 5 °C min^−1^ under an inert nitrogen atmosphere to avoid oxidation and continuing at 900 °C under an air atmosphere to promote oxidation.

#### 3.4.6. Rheology

Rheological properties of the gelating hydrogels were determined using a Physica MCR 101 Rheometer (Anton Paar, Graz, Austria), equipped with a cone-plate geometry (CP 25-1), with a constant gap of 0.048 mm and rugged plate-plate (PP25/S) 25 mm with a gap of 1 mm, allowing to control torques between 0.5 mN·m and 125 mN·m [62]. The cone-plate geometry was used for strain sweep and frequency sweep measurements, and the plate-plate geometry for measurements of temperature ramps. The linear viscoelastic range was determined by performing a strain sweep from 0.1 to 1000% at a constant angular frequency of 10 rad/s for 30% by weight of the different extraction methods of turbot gelatin hydrogels. The storage modulus G′ and loss modulus G″ were determined in the range of linear deformation. Frequency sweep measurements were also made from 0.05 to 600 rad/s applying a constant 0.1% strain. All experiments were carried out at 20 °C.

#### 3.4.7. Scanning Electron Microscopy

The morpho-structural features of turbot gelatin were examined through FEI Quanta 200 (FEI, Hillsboro, OR, USA)environmental variable electron microscope, with a working mode in High Vacuum (10^−5^ mbar), Under Vacuum (SM 6010 LA), an accelerating voltage of 20 kV in the electro-backscatter image, with a solid-state backscattered detector, an integrated energy dispersive X-ray spectroscopy (EDS) analyzer. For the preparation, the samples were covered with a layer of gold, approximately 15 nm thick.

### 3.5. Numerical Fitting and Statistical Analysis

Fitting procedures and parametric estimations calculated from the hydrolysis kinetics were carried out by minimizing the sum of quadratic differences between the observed and model-predicted values, using the non-linear least-squares (quasi-Newton) method provided by the macro-‘Solver’ of the Microsoft Excel spreadsheet. Confidence intervals from the parametric estimates (Student’s *t*-test) and consistency of mathematical models (Fisher’s F test) were evaluated by “SolverAid” macro (Levie’s Excellaneous website: http://www.bowdoin.edu/~rdelevie/excellaneous, accessed on 1 January 2015). The significance of comparisons between protocols was analyzed by ANOVA with a significance level of *p* < 0.05.

## 4. Conclusions

Extraction of gelatin from turbot skin and the subsequent production of collagen hydrolysates appears as a suitable strategy to recover valuable protein from filleting by-products. Of the various methods tested, sequential treatment of the skin with sodium hydroxide, sulphuric and citric acids at 22 °C allow to thermally extract the gelatin with the strongest rheological properties. These seem to correlate with structural integrity assessed by gel permeation chromatography (GPC), in particular with a high proportion of high molecular weight components and a low quantity of peptides below 100 kDa. Such Mw distributions probably reflect the differences seen by infrared spectroscopy in bands related to the secondary structure and random coil disposition of protein chains. In contrast to other studies, we found no relationship between rheological properties and amino acid composition. 

Turbot gelatin does not match the gel-forming and viscoelastic properties of gelatin from terrestrial animals, and therefore may not be suitable for the classical applications in food, pharma, cosmetics, and photographic industries, unless chemically modified to improve these characteristics. However, turbot gelatin shows potential in new applications, such as a food ingredient when fast dissolution in the mouth is desired [63] since gel-sol transition occurs close to 20 °C for the best performing gelatins. For the pharmaceutical industry, microencapsulation of liposoluble vitamins has been proposed with gelatins of up to 140 bloom [9], a range covered by turbot gelatin reported here.

Efficient hydrolysis of the remaining collagenous material can be achieved by proteolysis with alcalase, liquefying more than 90% of the original material into low molecular weight peptides. The amino acid profile, digestibility, and antihypertensive properties of these hydrolysates show their potential as food and feed ingredients.

## Figures and Tables

**Table 1:** Results of yield and gel strength (6.67% *w/v*) for the gelatin recovery from fresh skins of turbot under the various protocols of production tested. Values are average ± intervals of confidence for *n* = 2 (replicates of independent batches) and α = 0.05. Different letters in each file (as superscript) mean significant differences between methods (*p* < 0.05).

Method	Yield (%, w of Gelatin/w of Skin)	Gel Strength (g)
M1	5.22 ± 0.08 ^c^	177.0 ± 5.9 ^a^
M2	8.18 ± 0.09 ^a^	91.0 ± 21.6 ^c^
M3	4.82 ± 0.98 ^c^	132.0 ± 2.0 ^b^
M4	9.07 ± 1.26 ^a^	60.5 ± 16.7 ^c^
M5	5.20 ± 0.27 ^c^	81.5 ± 6.9 ^c^
M6	4.51 ± 0.51 ^c^	0.0 ± 0.0
M7	6.97 ± 0.61 ^b^	0.0 ± 0.0

**Table 2:** Molecular weight (kDa) of gelatin from turbot skin in distributions shown in Figure 1. Rt: retention time; Mw: weight average molecular weight; Mn: number average molecular weight; PDI: polydispersity index. Peak area (%) corresponds to refractive index detector. Values are represented as mean ± standard deviations (*n* = 2), except for M4.

Method	Peak Number	Rt (min)	Mw (kDa)	PDI	Peak Area (%)
**M1**	1-high Mw	37.6–44.3	-	-	7.9 ± 0.3
	2	45.3 ± 0.2	202.7 ± 0.5	1.044	31.5 ± 0.6
	3	48.4 ± 0.0	107.1 ± 2.6	1.023	32.0 ± 1,3
	4-low Mw	50.3–65.2	<100	-	28.6 ± 0.4
**M2**	1-high Mw	37.7–44.5	-	-	4.7 ± 0.2
	2	45.6 ± 0.0	208.1 ± 2.9	1.021	15.3 ± 0.9
	3	47.6 ± 0.0	150.3 ± 1.3	1.004	8.4 ± 018
	4	48.4 ± 0.0	111.6 ± 0.7	1.012	20.7 ± 0.9
	5-low Mw	48.4–67.4	<100	-	50.9 ± 3.7
**M3**	1-high Mw	36.9–44.7	-	-	3.8 ± 0.3
	2	45.6 ± 0.0	214.6 ± 4.3	1.024	11.5 ± 2.7
	3	47.5 ± 0.0	154.1 ± 1.8	1.004	6.1 ± 0.7
	4	48.4 ± 0.0	113.5 ± 0.7	1.011	21.7 ± 1.3
	5-low Mw	49.3–6.6	<100	-	57.0 ± 5.0
**M4**	1-high Mw	47.5	200.8	1.065	7.6
	2	48.9	104.5	1.021	20.7
	3-low Mw	49.9–70.0	<100	-	71.72
**M5**	1-high Mw	36.0–44.9	-	-	0.6 ± 0.0
	2	47.6 ± 0.1	200.4 ± 14.6	1.085	5.9 ± 4.2
	3	48.9 ± 0.4	113.1 ± 1.2	1.007	10.5 ± 3.1
	4-low Mw	49.1–69.8	<100	-	83.0 ± 7.4
**M6**	1-high Mw	37.0–44.6	-	-	1.1 ± 1.0
	2-low Mw	45.3–70.4	<100	-	98.9 ± 1.0
**M7**	1-high Mw	34.5–46.4	-	-	0.6 ± 0.2
	2-low Mw	45.7–70.3	<100	-	99.4 ± 0.2

**Table 3:** Assignments of IR bands to secondary structure of turbot gelatin.

Secundary Structure Elements	WaveNumber (cm^−^^1^)
β-turn/ β-sheet	1628–1632
Random Coil	1644–1650
Triple α-Helix	1660
β-turn/ β-sheet	1680–1690

**Table 4:** Results of SR hydrolysis by alcalase (CH1 and CH2) and papain (CH3 and CH4) in terms of mass balance, chemical characteristics, and composition, as well as bioactivities. Ydig: yield of digestion process. Yoil: percentage of oil recovered. Yskin: percentage of final solid produced (non-digestible skin). Pr: soluble protein by Lowry method. *Hm*: maximum hydrolysis degree from Weibull equation. *Hm*: maximum hydrolysis degree from Weibull equation. I_ACE_: maximum ACE activity. IC_50_: protein-hydrolysate concentration that generates 50% of I_ACE_. Different letters in each column (as superscript) mean significant difference between hydrolysates (*p* < 0.05).

	CH1	CH2	CH3	CH4
Mass balance and hydrolysates characteristics				
Y_dig_ (%)	91.0 ± 0.2 ^a^	93.1 ± 0.2 ^b^	77.0 ± 0.03 ^c^	77.1 ± 0.5 ^c^
Y_oil_ (%)	2.2 ± 0.1 ^a^	1.9 ± 0.2 ^a^	1.8 ± 0.2 ^a^	1.6 ± 0.6 ^a^
Y_skin_ (%)	1.9 ± 0.3 ^a^	1.8 ± 0.9 ^a^	9.0 ± 0.6 ^b^	7.5 ± 0.1 ^c^
Pr (g/L)	45.5 ± 0.6 ^a^	46.8 ± 0.4 ^b^	41.4 ± 0.3 ^c^	39.9 ± 0.4 ^d^
*H_m_* (%)	19.7 ± 0.3 ^a^	24.1 ± 0.3 ^b^	4.2 ± 0.5 ^c^	5.3 ± 0.1 ^d^
*v_m_* (% min ^−1^)	0.35 ± 0.02 ^a^	0.21 ± 0.01 ^b^	0.04 ± 0.01 ^c^	0.07 ± 0.00 ^d^
Amino acid composition of hydrolysates				
Gly (%)	11.9 ± 0.2 ^a^	11.1 ± 0.1 ^b^	13.8 ± 0.4 ^c^	12.5 ± 0.5 ^a^
Glu (%)	12.6 ± 0.4 ^a^	12.7 ± 0.7 ^a^	13.0 ± 0.5 ^a^	13.0 ± 0.4 ^a^
Pro (%)	7.5 ± 0.3 ^a^	7.1 ± 0.5 ^a^	8.3 ± 0.7 ^a^	7.6 ± 0.1 ^a^
OHPro (%)	4.0 ± 0.1 ^a^	4.7 ± 0.1 ^b^	4.8 ± 0.2 ^b^	4.6 ± 0.1 ^b^
TEAA/TAA (%)	38.3 ± 0.4 ^a^	39.2 ± 0.4 ^b^	35.1 ± 0.4 ^c^	37.4 ± 2.1 ^a^
Antihypertensive and digestibility properties				
Dig (%)	90.3 ± 0.6 ^a^	94.2 ± 0.7 ^b^	82.3 ± 1.0 ^c^	86.3 ± 0.8 ^d^
I_ACE_ (%)	69.5 ± 3.5 ^a^	88.1 ± 1.8 ^b^	52.1 ± 4.7 ^c^	65.0 ± 0.7 ^d^
IC_50_ (µg Pr/mL)	131.2 ± 8.5 ^a^	40.3 ± 3.3 ^b^	976.1 ± 24.2 ^c^	462.1 ± 12.5 ^d^

## Data Availability

Not applicable.

## Supplementary Materials

The following are available online at https://www.mdpi.com/article/10.3390/md19090491/s1, Table S1: Amino acid (AA) content of gelatin recovered from fresh turbot skins (% or g/100 g total amino acids) with each protocol of production. OHPro: hydroxyproline. Pr: % of protein present, as the sum of amino acids, in the extracted gelatin sample. TE/TA: ratio total essential amino acids for human/total amino acids. Errors are the confidence intervals for *n* = 2 (replicates of independent batches) and α = 0.05; Table S2. Identification of bands by FTIR corresponding to Turbot gelatin; Table S3. The molecular weight (kDa) of hydrolysates from turbot skin after gelatin extraction is shown in Figure 8. Rt: retention time; Mw: weight average molecular weight; Mn: number average molecular weight; PDI: polydispersity index. Values are represented as mean ± standard deviations (*n* = 2); Figure S1. Flow chart of the methods applied to turbot skin for gelatin extraction.
