# Supplementary material for: Production and Physicochemical Characterization of Gelatin and Collagen Hydrolysates from Turbot Skin Waste Generated by Aquaculture Activities
Source: Mar Drugs. 2021 Aug 28;19(9):491. doi: 10.3390/md19090491 (PMC8465087; doi:10.3390/md19090491)
Supplement: Supplementary file 1 [file marinedrugs-19-00491-s001.zip › marinedrugs-1351781 sup_mat.pdf]

Table S1. Amino acid (AA) content of gelatin recovered from fresh turbot skins (% or g/100 g total amino acids) with each protocol of production. OHPro: hydroxyproline. Pr: % of protein present, as the sum of amino acids, in the extracted gelatin sample. TE/TA: ratio total essential amino acids for human/total amino acids. Errors are the confidence intervals for n=2 (replicates of independent batches) and α=0.05.

AA	M1	M2	M3	M4	M5	M6	M7
**Asp**	5.76±0.02	5.76±0.14	5.69±0.10	5.76±0.02	6.05±0.17	6.51±0.15	6.02±0.01
**Thr**	2.50±0.03	2.49±0.06	2.47±0.07	2.51±0.03	2.60±0.11	2.53±0.11	2.52±0.03
**Ser**	5.55±0.12	5.51±0.12	5.64±0.11	5.56±0.03	6.02±0.31	6.53±0.05	5.53±0.06
**Glu**	10.22±0.51	10.12±0.27	10.45±0.08	10.30±0.18	10.57±0.27	10.18±0.09	10.39±0.06
**Gly**	21.68±0.12	21.60±0.66	22.11±0.46	21.70±0.26	20.91±0.03	21.53±0.73	21.07±0.09
**Ala**	10.80±0.38	10.84±0.19	10.98±0.04	10.92±0.23	10.66±0.07	10.53±0.16	10.65±0.05
**Cys**	0.35±0.01	0.37±0.05	0.34±0.03	0.35±0.00	0.40±0.07	0.37±0.02	0.31±0.00
**Val**	1.76±0.06	1.77±0.04	1.80±0.07	1.83±0.02	1.77±0.16	1.80±0.08	1.74±0.07
**Met**	2.35±0.13	2.38±0.21	2.43±0.04	2.46±0.06	2.40±0.00	2.28±0.03	2.33±0.06
**Ile**	0.84±0.05	0.82±0.02	0.86±0.03	0.87±0.05	0.81±0.02	0.85±0.02	0.86±0.00
**Leu**	2.51±0.02	2.51±0.05	2.59±0.13	2.68±0.09	2.41±0.06	2.53±0.07	2.51±0.08
**Tyr**	0.84±0.05	0.88±0.05	0.83±0.01	0.79±0.04	0.91±0.09	1.39±0.04	0.89±0.05
**Phe**	2.68±0.06	2.75±0.13	2.67±0.09	2.63±0.07	2.74±0.19	2.81±0.00	2.76±0.00
**His**	0.76±0.05	0.77±0.06	0.72±0.01	0.74±0.10	0.75±0.00	0.75±0.13	0.80±0.03
**Lys**	3.89±0.17	3.92±0.02	3.87±0.06	4.03±0.01	3.78±0.26	3.98±0.05	4.00±0.14
**Arg**	8.10±0.08	8.23±0.61	7.78±0.16	7.95±0.03	8.08±0.11	8.10±0.13	8.09±0.08
**OHPro**	8.40±0.70	8.17±0.79	7.85±0.17	7.89±0.35	8.11±0.45	7.31±0.34	7.38±0.29
**Pro**	11.03±0.23	11.11±0.07	10.90±0.16	11.01±0.12	11.03±0.42	10.02±0.05	10.16±0.30
**Pr (%)**	94.9±1.6	94.6±1.4	94.7±1.2	93.4±0.9	95.1±0.8	91.0±0.8	89.8±1.0
**TE/TA (%)**	25.4±0.1	25.6±0.3	25.2±0.7	25.7±0.3	25.1±0.7	25.6±1.3	25.6±1.2

Table S2. Identification of bands by FTIR corresponding to Turbot gelatin

M1	M2	M3	M5	Description
3286	3277	3277	3277	Stretching of N-H bonds of protein, O-H groups of carbohydrates and water
3071	3070	3071	3076	N-H stretching
2850-3000	2850-3000	2850-3000	2850-3000	Tension modes, C-H bonds of aliphatic chains
1355/1445	1335/1446	1335/1445	1334/1446	Flexion C-H
1631	1628	1627	1628	Amide I
1520	1520	1531	1528	Amide II
1236	1237	1238	1236	Amide III
550	540	551	548	Amide IV
604	602	600	654	Amide V
702	702	708	700	Amide VI
923	923	926	924	Symmetric tension mode CNC bond
1161/1077/1031	1163/1077/1030	1163/1077/1030	1163/1077/1030	C-O<br>C-O-C

*Values in cm^{-1}

Table S3. Molecular weight (kDa) of hydrolysates from turbot skin after gelatine extraction shown in Figure 8. Rt: retention time; Mw: weight average molecular weight; Mn: number average molecular weight; PDI: polydispersity index. Values are represented as mean ± standard deviations (n=2).

Sample	Enzyme	Time	Mw (kDa)	Mn (kDa)	PDI
**CH1**	Alcalase	2 h	1376 ± 115	907 ± 83	1.517
**CH2**		4 h	1040 ± 45	730 ± 15	1.425
**CH3**	Papain	2 h	9975 ± 181	4359 ± 95	2.288
**CH4**		4 h	8687 ± 558	3453 ± 450	2.516

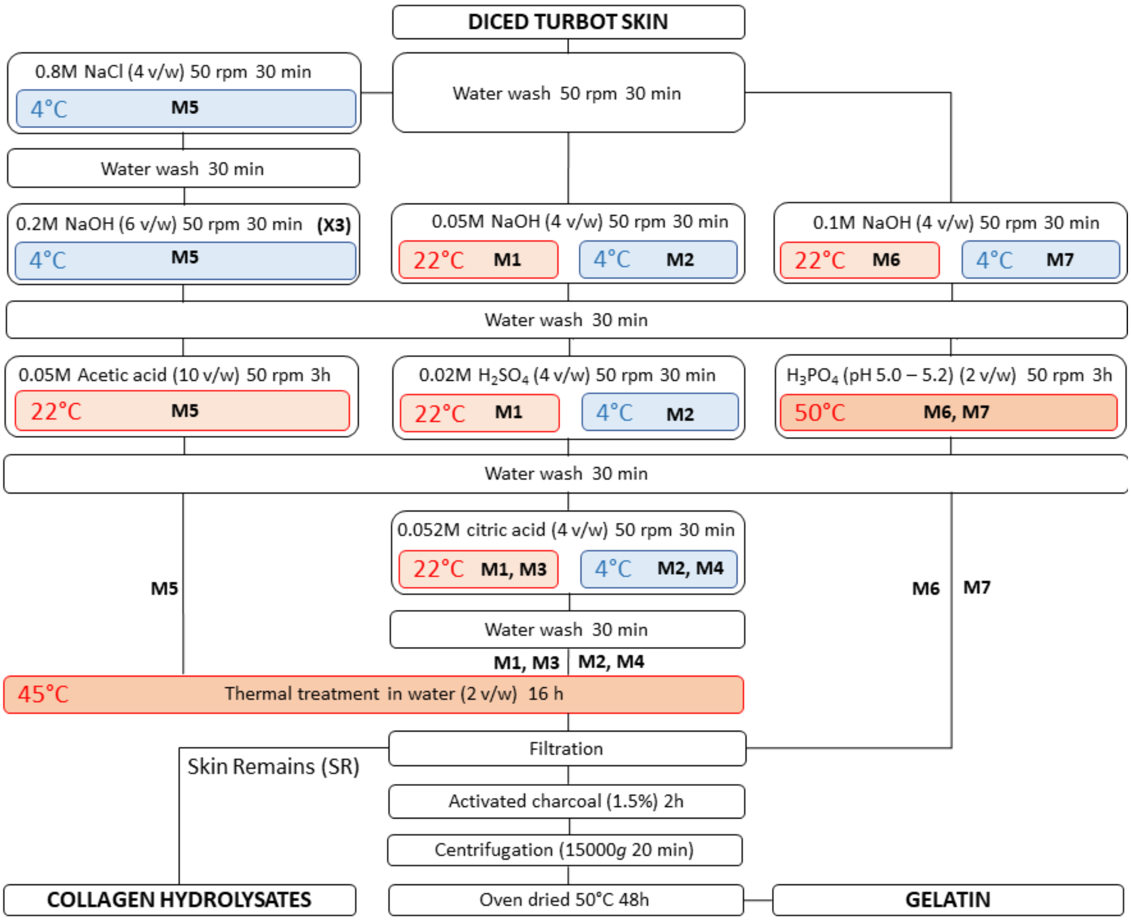

Figure S1. Flow chart of the methods applied to turbot skin for gelatine extraction.
